# Biomaterials for the Prevention of Oral Candidiasis Development

**DOI:** 10.3390/pharmaceutics13060803

**Published:** 2021-05-27

**Authors:** Dan Cristian Gheorghe, Adelina-Gabriela Niculescu, Alexandra Cătălina Bîrcă, Alexandru Mihai Grumezescu

**Affiliations:** 1 “Carol Davila” University of Medicine and Pharmacy, 050474 Bucharest, Romania; gheorghe.dancristian@gmail.com; 2 “M.S. Curie” Clinical Emergency Hospital for Children, 077120 Bucharest, Romania; 3 Faculty of Engineering in Foreign Languages, University Politehnica of Bucharest, 060042 Bucharest, Romania; niculescu.adelina19@gmail.com; 4 Faculty of Applied Chemistry and Materials Science, University Politehnica of Bucharest, 060042 Bucharest, Romania; ada_birca@yahoo.com or; 5 Research Institute of the University of Bucharest—ICUB, University of Bucharest, 050657 Bucharest, Romania

**Keywords:** fungal infections, oral candidiasis, antifungal drugs, anti-*Candida* compounds, antifungal biomaterials

## Abstract

Thousands of microorganisms coexist within the human microbiota. However, certain conditions can predispose the organism to the overgrowth of specific pathogens that further lead to opportunistic infections. One of the most common such imbalances in the normal oral flora is the excessive growth of *Candida* spp., which produces oral candidiasis. In immunocompromised individuals, this fungal infection can reach the systemic level and become life-threatening. Hence, prompt and efficient treatment must be administered. Traditional antifungal agents, such as polyenes, azoles, and echinocandins, may often result in severe adverse effects, regardless of the administration form. Therefore, novel treatments have to be developed and implemented in clinical practice. In this regard, the present paper focuses on the newest therapeutic options against oral *Candida* infections, reviewing compounds and biomaterials with inherent antifungal properties, improved materials for dental prostheses and denture adhesives, drug delivery systems, and combined approaches towards developing the optimum treatment.

## 1. Introduction

The oral microbiota is a normal part of the oral cavity, including several hundred to several thousand different microorganisms. Its role is to protect against colonization of extrinsic infectious agents, which could affect the overall health [1]. However, under certain circumstances, oral infections can occur. Poor oral hygiene, malnutrition, use of antibiotics, trauma, endocrinopathies, and use of removable prosthesis are only a few of the factors that favor infections by invasive fungal pathogens [2,3,4].

*Candida* species represent a class of such pathogens. In healthy individuals, *Candida* is a harmless organism found in the mucous membranes such as ears, eyes, nose, mouth, gastrointestinal tract, reproductive organs, and skin. In immunocompromised patients, it becomes overgrown, causing opportunistic infections, with symptoms varying from mild localized rashes to severe disseminated infections. *Candida* infections are known as candidiasis (sometimes found in the literature as candidosis), the *Candida* infections localized in the oral cavity being generally termed oral candidiasis [4,5,6,7,8].

Oral candidiasis affects the intraoral, pharyngeal, and perioral regions, being a frequent source of discomfort, pain, loss of taste, and aversion to food [9,10]. Moreover, when the immune system is weakened or a disruption in the host environment, there is a risk of tracheal or esophageal extension and even systemic dissemination, which may be life-threatening [6,11,12].

Oral candidiasis treatment consists of the administration of conventional antifungal agents. Nonetheless, the efficiency of this approach is impaired by the emergence of drug-resistant *Candida* species. Hence, new therapeutic strategies have to be developed [7].

In this respect, this paper reviews oral candidiasis from the perspectives of causative pathogens, risk factors, and classic treatment options, further focusing on novel alternatives against *Candida* infections. Specifically, there are extensively described intrinsic anti-*Candida* compounds and biomaterials, replacements for classic prostheses and adhesives materials, controlled release drug-delivery systems, and combinations of these strategies to obtain optimum treatment.

This review aims to thoroughly present the state of the art of oral candidiasis to set a clear context for future research. Explicitly, through a deep understanding of the current and developing treatment options, better solutions can be envisaged for preventing the overgrowth of drug-resistant *Candida* species.

## 2. Causative Pathogens and Risk Factors

**Figure 1:**
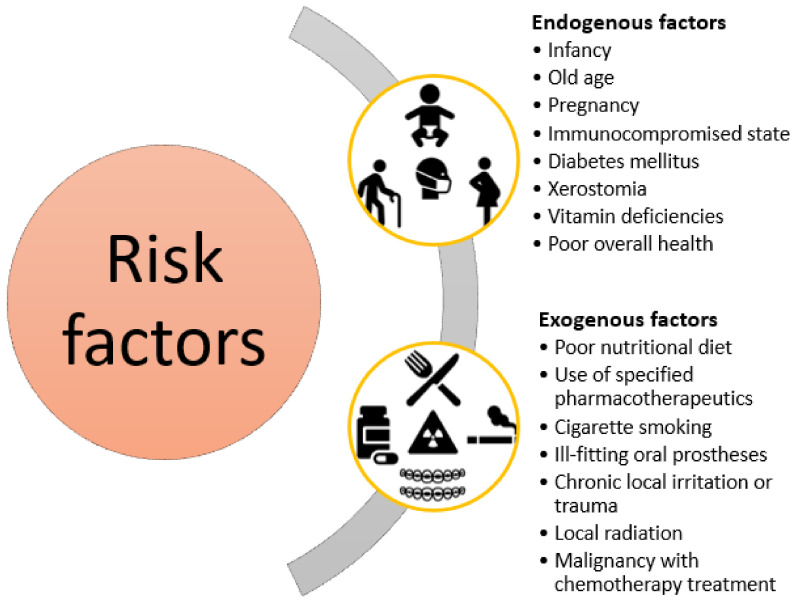
Classification of risk factors associated with oral candidiasis development. Created based on information from literature references [6,10,17,18,19,20].

*Candida* is a genus of yeast fungus belonging to the division Ascomycota [13] that can exist both as a commensal organism and an opportunistic pathogen in the human body [10,14,15,16]. *Candida* species are normal microbiota components of the mucosal oral cavity, gastrointestinal system, and genitourinary tracts [17]. When there is an imbalance in the normal oral flora, the overgrowth of *Candida* spp. may occur, thus producing oral candidiasis [5]. *Candida* spp. are present as yeasts in a healthy state, but under certain conditions, may transform into a pathogenic hyphael form [10]. The predisposing factors of oral candidiasis development include immune dysfunctions, immune suppressant drugs, prolonged antibiotic therapy, xerostomia, diabetes, human immunodeficiency virus (HIV) infection, chemotherapy, radiotherapy, alcohol and tobacco use, and dental prostheses [10,17,18,19] (Figure 1).

*Candida* is one of the most common causes of fungal infections worldwide, being responsible for more than 400,000 infections annually [21]. The incidence of candidiasis has increased recently due to the aging population and growing numbers of immunocompromised patients [22].

Out of the *Candida* species, *Candida albicans* is considered the primary causative pathogen of oral candidiasis [14,17,23]. This is due to its high capability of adherence to oral tissues and denture surfaces, resulting in biofilm formation [14,24]. *C. albicans* is also the most virulent pathogenic *Candida* species, accounting for 70–80% of isolates from oral mucosal lesions [25].

**Figure 2:**
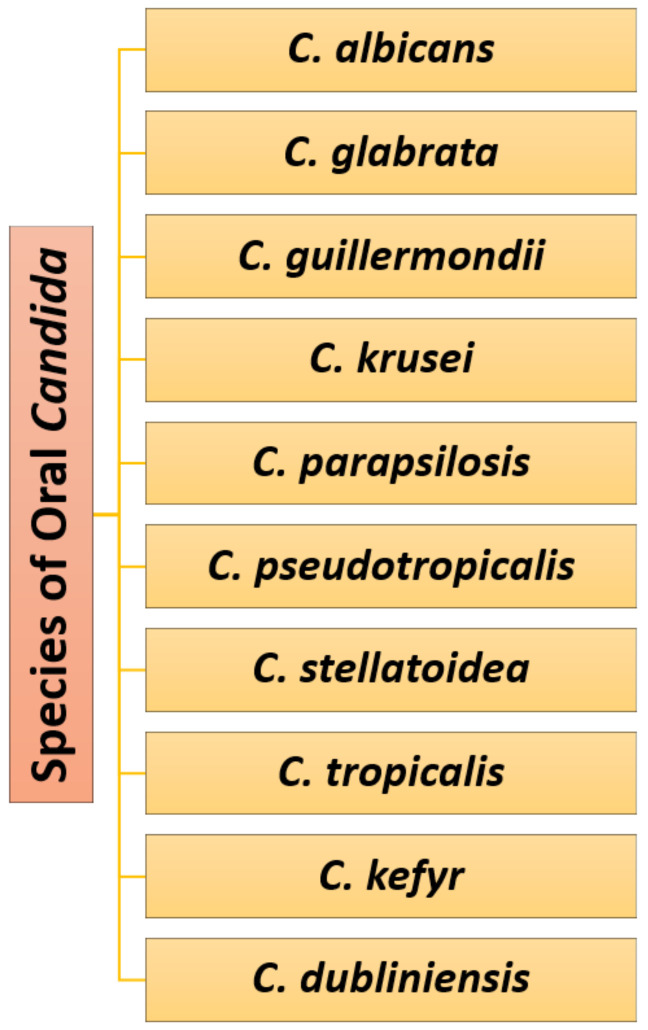
*Candida* spp. causing oral candidiasis. Created based on information from literature references [5,10,14,17,22,26,27,28].

Oral candidiasis can be also caused by non-*albicans Candida* species. Microorganisms like *C. glabrata*, *C. guillermondii*, *C. krusei*, *C. parapsilosis*, *C. pseudotropicalis*, *C. stellatoidea*, *C. tropicalis*, *C. keyfr*, and *C. dubliniensis* are also responsible for oral infections, becoming more prevalent and important opportunistic pathogens in immunocompromised patients [5,14,22,26,27,28,29] (Figure 2). Moreover, some of these species have intrinsic resistance to antifungals (e.g., *C. glabrata* and *C. krusei*) and/or rapidly develop such resistance (e.g., *C. parapsilosis* and *C. tropicalis*) [12,30].

Identifying the responsible pathogen for the infection is essential for choosing the best-suited antifungal agent, as susceptibility to different drugs varies between *Candida* species (Table 1).

Besides, *Candida* spp. may further interact with various microorganisms within the mouth, leading to a complex and mixed biofilm with an organized structure that is difficult to remove [24]. Pathogens accumulation on the host’s mucous membranes, acrylic surfaces of removable orthodontic devices, and denture prostheses results in the production of proteolysis enzymes that damage mucosal cells [18]. Hence, there is created a dangerous focus of inflammation that increases the risk of cerebral strokes, decompensated glycemia, and focal and autoimmune diseases [17]. Coupled with their drug resistance, biofilms lead to challenges in developing therapeutic approaches to prevent and cure oral infections [13].

Severe fungal infections have been especially reported in HIV infected individuals, patients undergoing hematopoietic stem cell transplantation, and those receiving intensive chemotherapy and radiotherapy. In particular, the latter factors facilitate fungal overgrowth as they modify the physiology and microbial ecology of the oral environment to prolonged xerostomia and hyposalivation. Moreover, due to the compromised immune defense mechanisms, systemic infections may occur, thus resulting in significant patient morbidity [29]. To avoid infection generalization, prophylaxis treatment against *Candida* can be provided to predisposed patients. However, it must be proceeded with care as, in hematological malignancies and stem cell transplant recipients, a microbiota imbalance may occur, and *Aspergillus* and other molds may overgrow to produce dangerous fungal infections instead [31].

## 3. Classic Treatment Options

In denture wearers, oral candidiasis’ current management relies on good hygiene practices, close attention to proper denture fit with tissue conditioners/liners/rebases, and administration of antifungal drugs [20]. Immunocompetent patients respond well to topical or oral medications, but there is a high risk of systemic infection in the case of the elderly and medically or immunologically compromised patients [4].

**Figure 3:**
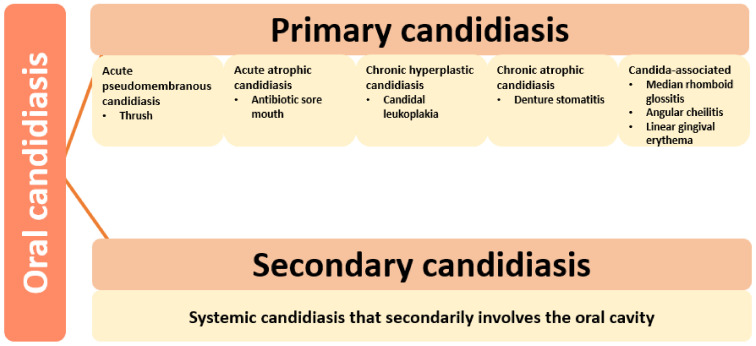
Types of oral candidiasis. Created based on information from literature references [6,14,32].

Depending on the affected tissues, oral candidiasis can be classified into primary and secondary. Primary candidiasis refers to infections that only involve oral or perioral tissues, while secondary candidiasis is a systemic *Candida* infection that collaterally affects the oral cavity [6]. Based on the clinical manifestations, primary oral candidiasis can be further divided into several subclasses, as presented in Figure 3.

Depending on the type of oral candidiasis, several treatment options can be employed (Table 2). The most conventional and efficient currently available drugs for treating oral candidiasis are polyenes (e.g., amphotericin B and nystatin), azoles (e.g., miconazole, clotrimazole, fluconazole, itraconazole, voriconazole, posaconazole, and ketoconazole), and echinocandins (e.g., anidulafungin, caspofungin, and micafungin). These antifungal agents can be administered either locally or systemically, in various forms ranging from oral suspensions, ointments, creams, gels, and troches, to tablets, pastilles, and even intravenous infusions. However, due to their toxicity, adverse side effects, and acquired resistance, these therapeutics action is often hindered [7,8,15,19,22,27,33,34,35].

Conventional local oral delivery formulations usually exhibit an initial burst release that rapidly decreases to subtherapeutic concentrations [8], whereas regular antifungal systemic drugs result in severe side effects [48]. Therefore, novel treatment options must be considered for improving anti-*Candida* medicine efficiency while protecting the organism from potentially harmful effects.

An alternative to medication is the use of antiseptic mouthwashes for preventing oral candidiasis development [49]. Their inclusion in oral hygiene practices helps avoid excessive colonization of fungal pathogens and delay *Candida* biofilm formation. Particularly, mouthwashes containing cetyl pyridinium chloride or chlorhexidine were shown effective against both planktonic and biofilm embedded fungal cells [50].

## 4. Novel Treatment Options

**Figure 4:**
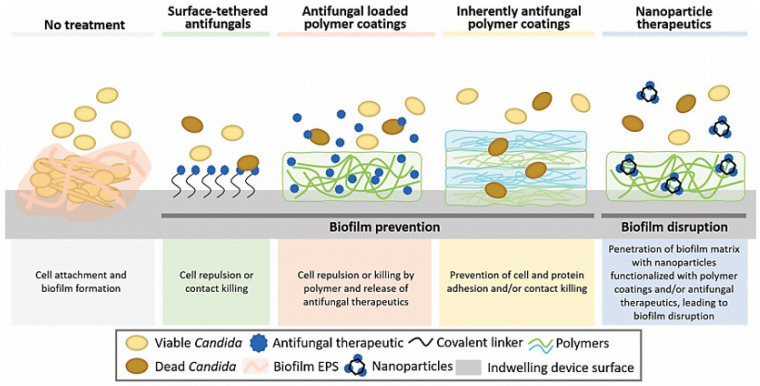
Schematic representation of different biomaterial strategies to combat surface-associated *Candida* biofilms. Reproduced from [21], Frontiers in Microbiology, 2020.

As oral candidiasis’ current treatment is becoming rather ineffective due to the emergence of resistant strains, there is an increased research interest towards novel treatment options. The investigated strategies include the use of intrinsic anti-*Candida* materials, antimicrobial nanoparticles, and natural antifungal essential oils and extracts, replacing traditional prosthesis materials and denture adhesives with biomaterials capable of preventing biofilm formation, including regular antifungal agents into targeted and controlled release delivery systems, and combined approaches towards developing the optimum treatment [3,4,48,51,52,53] (Figure 4).

### 4.1. Intrinsic Anti-Candida Biomaterials/Compounds

Several materials inherently have antifungal properties that can be exploited in developing superior treatments for oral candidiasis. In this respect, polymeric materials, inorganic nanoparticles, and natural products with intrinsic anti-*Candida* activity are further discussed.

#### 4.1.1. Polymeric Materials

Chitosan is a natural polymer possessing several beneficial properties, such as biodegradability, biocompatibility, fungicidal, antimicrobial, and antitumor activities [54,55,56,57]. It is considered a promising component of mouthwashes and denture adhesives for preventing oral candidiasis [55]. Moreover, low-molecular-weight chitosan solution can be effectively used as an antifungal denture cleanser, showing a significant reduction in *C. albicans* viability in biofilms formed on polymethyl methacrylate [58]. Recently, Ikono et al. [59] examined chitosan nanoparticles of 20–30 nm in diameter for their ability to inhibit *C. albicans* biofilm growth following initial cell attachment. After 3 h of incubation, a greater than 50% reduction in biofilm mass was reported, concluding that chitosan nanoparticles possessed inherent antibiofilm activity but could not entirely inhibit or disrupt *Candida* biofilms [21].

Nylon-3 polymers have been proven to have significant activity against pathogenic strains of *C. albicans* that are resistant to conventional medication. Particularly, nylon-3 polymers with β-amino residues (βNM) in their backbone structure attracted more interest due to their resemblance to proteins that induce biocompatibility [60,61,62,63]. Moreover, such nylon-3 polymers can be easily prepared, being promising as clinical antifungal agents [60]. Liu et al. [60] have reported that poly-βNM with 20-mer average length displayed strong and selective activity against *C. albicans* strain K1, while only very little hemolysis or toxicity toward 3T3 fibroblasts was detected. Rank et al. [62] have also researched the antifungal activity of nylon-3 polymers. They have evaluated the action of a host defense peptide-like nylon-3 copolymer, obtaining efficacy levels comparable to those of amphotericin B and fluconazole, displaying only mild to moderate toxicity toward mammalian cells.

Guanidines are another class of cationic polymers that can be used as antiseptics and antimicrobials. Particularly, polyhexamethylene guanidine hydrochloride (PHMGH) was evaluated for its antifungal properties [61,64,65,66]. Choi et al. [67] reported a more potent antifungal activity of PHMGH than amphotericin B, with no hemolytic and lactate dehydrogenase release activities. The researchers also investigated the mechanism of action against *C. albicans*, proving that PHMGH exerts its fungicidal effect by forming pores in the cell membrane. Martini Garcia et al. [64] tested an aqueous solution containing PHMGH against mature *Candida* biofilms formed on denture liner specimens. They registered a total fungal elimination after 10 min of contact without affecting the mechanical properties of the denture liners.

#### 4.1.2. Inorganic Nanoparticles

Silver nanoparticles (AgNPs) are some of the most studied inorganic nanoparticles, being widely utilized for their antimicrobial activity [68,69]. Due to their unique physicochemical properties, beneficial interactions with living structures, and nontoxicity for healthy human tissues, AgNPs may represent key components in developing novel biomedical strategies [21,70,71,72,73,74,75,76,77]. Researchers have reported considerable antifungal activity against *Candida* spp., with potent antibiofilm and cell disruption ability [13,27,78,79,80]. Monteiro et al. [81] indicated that AgNPs could be used in the treatment of denture stomatitis. The researchers noticed a higher antifungal activity against *C. glabrata* than against *C. albicans*, and more effective action in inhibiting *Candida* biofilm formation than in controlling mature biofilms.

Selenium nanoparticles (SeNPs) have recently gained attention for their antimicrobial properties [21,82]. Shakibaie et al. [83] have demonstrated the anti-*Candida* effects of nanoscale biogenic elemental Se, stating that the mechanism of action requires additional investigation. Guisbiers et al. [84] have synthesized pure SeNPs that successfully inhibited *C. albicans* biofilm formation by adhering to the biofilm, penetrating into the pathogen, and consequently damaging the cell structure by substituting sulfur with selenium. These nanoparticles were able to reduce by 50% the fungal burden in mature biofilms at a concentration of only 25 ppm.

Several nanoscale metal oxides have also been observed to have antifungal properties. Nanoparticles of iron oxide [85,86,87,88], zinc oxide [89,90,91,92,93], magnesium oxide [93,94,95,96], calcium oxide [94,97], copper oxide [98,99,100], titanium dioxide [101,102,103,104], bismuth oxide [105,106], and silver oxide [107] display fungistatic and/or fungicidal activities that are useful in the treatment and prevention of oral candidiasis.

#### 4.1.3. Natural Products

Natural products represent a great source of various useful chemical compounds that can be included in diverse biomedical applications [108]. Recently, there is an increased interest in using natural essential oils and extracts as a safer and more efficient alternative to classic antifungal drugs [18,109,110].

Basil extracts have potential use against *Candida* spp. [111,112]. Roozbehani et al. [18] evaluated the effect of basil extracts on *C. albicans* and *C. dubliniensis* adhesion to acrylic surfaces of removable orthodontic appliances. The researchers concluded that such extracts could inhibit the growth, adherence, and formation of biofilms, having great potential as antifungal solutions or mouthwashes.

*Equisetum giganteum*, popularly known as ‘horsetail’ is another plant of antifungal importance [113,114,115]. Martins Almeida et al. [116] have incorporated *E. giganteum* hydroethanolic extracts into denture adhesives, interfering in the development of *C. albicans* biofilm. This plant extract significantly minimized pathogen colonization and reduced its metabolism, being a promising solution for treating and preventing denture stomatitis.

*Coriandrum sativum* essential oil has also been shown to have inhibitory effects on *Candida* spp., acting similarly to nystatin and amphotericin B [110,117,118]. The results obtained by Furletti et al. [119] indicate the potential use of crude *C. sativum* oil in the prevention and treatment of oral candidiasis, as it demonstrated strong activity against both *Candida* spp. planktonic cells and *C. albicans* biofilm.

Curcumin is an important compound that can be extracted from turmeric [120]. Its antifungal activity is exhibited through various mechanisms, such as targeting metabolic paths, inducing apoptosis, and increasing reactive oxygen species. These properties of curcumin are effective in the design of drug formulations with fewer side effects and superior performance [53,121,122,123,124]. Narayanan et al. [125] have evaluated curcumin’s inhibitory action against *C. albicans*, *C. parapsilosis*, *C. glabrata*, and *C. dublieniensis*, proving its potential as a therapeutic alternative to conventional antifungals.

Cinnamon essential oils, cinnamon extracts, and pure compounds also show significant antimicrobial activities against oral pathogens [126]. The antifungal properties of cinnamon are more pronounced than its antibacterial activity, indicating potential use in candidiasis treatments either as the main or a complementary agent [127,128,129,130]. De Araujo et al. [131] analyzed the efficacy of mouthwash and spray containing essential oil of *Cinnamomum zeylanicum* Blume for the treatment of oral candidiasis. A mycological analysis demonstrated a reduction of 61% and 33% of *Candida* spp., isolated from oral mucosa and dentures, respectively. *C. tropicalis* elimination was reported in both sites.

Propolis is another natural product presenting anti-*Candida* activity [9]. Ota et al. [132] performed an in vivo study on patients with full dentures who used a hydroalcoholic extract of propolis as a mouth-rinse. The researchers studied the antifungal activity of propolis by sensitivity tests on 80 strains of *Candida* yeasts (20 strains of *C. albicans*, 20 strains of *C. tropicalis*, 20 strains of *C. krusei*, and 15 strains of *C. guilliermondii*). A clear antifungal activity was reported, with the order of sensitivity *C. albicans* > *C. tropicalis* > *C. krusei* > *C. guilliermondii*. Siquiera et al. [9] have also reported the susceptibility to red propolis alcoholic extract of *C. albicans*, *C. tropicalis*, and *C. glabrata* isolated from chronic periodontitis cases.

*Camellia sinensis* and *Hypericum havvae* possess exceptional anti-*Candida* properties and can be used for developing alternative antifungal medication. *Camellia sinesis* has been shown effective against *C. albicans*, *C. parapsilosis*, *C. tropicalis*, and *C. glabrata*, while *Hypericum havvae* is a promising agent against *C. glabrata*, *C. kreusei*, *C. parapsilosis*, *C. guilliermondii*, and *C. tropicalis* [133].

### 4.2. Biomaterials for Oral Prosthesis and Denture Adhesives

*Candida* spp. have been shown to form biofilms on the surface of various medical devices made of PMMA, silicone elastomer, polyurethane, polyvinyl chloride, polypropylene, and polystyrene, among others [27]. Additionally, the use of denture adhesives, besides their functional and psychological advantages, has been reported to predispose wearers to oral candidiasis [134]. Hence, these two elements could hold great improvement potential in synergy with fungicidal or fungistatic materials [135].

By coating or functionalizing currently used materials, the oral prosthesis can inherit antifungal properties. As PMMA is one of the most commonly used polymers for fabricating a broad range of dental appliances, most of the studies found in the literature focus on enhancing this material’s biocompatibility and functionality [27,48,136,137,138,139,140,141].

Jung et al. [20] have reported a novel fungal repelling multilayer coating for PMMA-based denture materials. The researchers created an alternating structure through layer-by-layer (LBL) self-assembly. Specifically, amphiphilic quaternary ammonium chitosan was employed as the positive antimicrobial layer, whereas sodium alginate was used as the negative layer to create LBL multilayers on the substrate material. The final composite material was shown to be biocompatible toward mammalian cells and resist under shaking and repeated brushing, indicating a novel long-term strategy in controlling fungal biofilms formation on denture biomaterials.

Different attempts have been made aiming to modify and improve the mechanical properties of PMMA by incorporating various metal oxide fillers and fibers [139]. For instance, studies have proven that adding zirconia nanoparticles (ZrO_2_ NPs) to PMMA denture base increases the density and reduces porosity, leading to enhanced flexural strength, tensile strength, and fracture toughness [48]. Moreover, Gad et al. [142] have demonstrated that the addition of ZrO_2_ NPs to cold-cured acrylic resin reduces *Candida* adhesion due to its denser and less porous lattice. Gowri et al. [143] have attributed the inhibitory activity of ZrO_2_ NPs against fungal strains to their interference in cell function and resulting deformation in fungal hyphae. Hence, these nanoparticles could be included in the material for repairing denture bases and in the PMMA removable prostheses as a possible strategy for preventing denture stomatitis.

Mahmudi et al. [144] proposed the addition of nano-zirconia into the denture adhesive instead of the base material. The researchers observed *C. albicans* growth inhibition at concentrations higher than 31 μg/mL. Therefore, ZrO_2_ NPs can be added to the denture adhesives to reduce the possible occurrence and reduce the incidence of *C. albicans*. However, the formulation is effective for prevention purposes only, as it did not cause pathogens death. Alternatively, Namangkalakul et al. [22] stated that high-molecular-weight water-soluble chitosan can serve as an antifungal adhesive to prevent and treat denture stomatitis. Besides, denture adhesives could be used as delivery systems for antifungal agents without affecting their adhesion capacity [134].

A significant reduction of *C. albicans* adherence was also noticed in PMMA imbedded with spherical Ag NPs. Acosta-Torres et al. [140] have evaluated the flexural properties of PMMA-Ag NPs material, showing that they fit within standard required values. Moreover, the obtained biomaterial can be used as a biocompatible antifungal PMMA denture base material that does not affect metabolism and proliferation and does not cause genotoxic damage to cells. A similar strategy was approached by Nam et al. [145]. When combined with Ag NPs at 20 wt %, the researchers reported that the resin displayed antifungal activity while maintaining appropriate physical properties. Nonetheless, it was concluded that color stability must be improved for clinical use.

PMMA behavior can also be improved by the addition of zinc oxide nanoparticles (ZnO NPs). In this respect, Cierech et al. [138] have reported a four-fold higher inhibitory activity on *C. albicans* growth for a 7.5% concentration of ZnO NPs, evaluating the efficacy of nanocomposites PMMA-ZnO-NPs and sputtered ZnO nanoparticles on the PMMA layer. The mechanism through which the antifungal effect is exerted is not completely understood, but it is supposed to happen due to increased concentration of intracellular singlet oxygen, leading to oxidative stress. Kamonkhantikul et al. [141] have also made use of ZnO NPs. The researchers evaluated antifungal, optical, and mechanical properties of heat-cured PMMA incorporated with various amounts of ZnO NPs with or without methacryloxypropyltrimethoxysilane modification. At the same concentration of ZnO NPS, silanized groups resulted in a greater reduction in *C. albicans* than the non-silanized ones. The best outcomes were reported for PMMA incorporated with 2.5% silanized ZnO NPs, which showed greater antifungal activity, less color difference, and opacity than non-silanized nanoparticles while preserving the mechanical properties of the base material.

Another interesting approach to modifying PMMA is to reinforce this polymer with nanodiamond (ND). Mangal et al. [139] have reported significant improvement in the mechanical properties of PMMA with the incorporation of as little as 0.1 wt% ND resulting in a more than 20% increase in flexural strength over unmodified PMMA. Moreover, the researchers observed pronounced resistance to *C. albicans* and a significant reduction in the formation of salivary biofilm.

### 4.3. Drug Delivery Systems

As several commonly used antifungal drugs present limited water solubility, poor oral bioavailability, and limited formulation approaches, there is a strong need to develop innovative drug delivery systems [146].

For the treatment of oral candidiasis, sustained drug release is required so that the medication is retained in the oral cavity and produces an antifungal effect for a prolonged time [8]. One patient-friendly option is to elute drugs from biomaterials in order to treat and prevent the fungal infections associated with the use of dental prostheses [27]. In this regard, nanofiber-based scaffolds have recently become popular due to their remarkable properties such as low density, large specific surface areas, high porosity, and very small pore sizes [8].

Another promising possibility is to use nanoparticles for drug delivery, as they improve the biopharmaceutical and pharmacokinetic properties of antifungal agents. These characteristics are further reflected in a greater pharmacodynamic potential, lower toxicity, and prolonged action [147]. In particular, polymeric nanoparticles are attractive due to their two-fold role: drug nanocarrier and intrinsic antimicrobial agent [52]. As reported in the literature, biodegradable polymers such as poly-lactic-glycolic acid (PLGA), chitosan, and liposomes promote a slow, sustained drug release, thus diminishing the medicine dosage and its associated toxicity. Hence, adverse effects are reduced without compromising the therapeutic fungicidal action [15].

Lipid-based nanoparticles are also promising moieties for penetrating the biofilm matrix and targeting fungal cells [21]. Al-Maghrabi et al. [148] have successfully encapsulated miconazole into solid lipid nanoparticles (SLNs). According to the researchers, the susceptibility of *C. albicans* to miconazole-loaded SLNs using a well-diffusion technique indicated that the antifungal activity was enhanced when incorporated into the SLNs. Similarly, lipid-based formulations of amphotericin B showed a significant decrease in side effects (i.e., nephrotoxicity) while preserving its broad-spectrum antifungal activity [146].

### 4.4. Combined Approaches

Regardless of their individual efficacy, the above presented biomedical strategies work best in synergy. In this regard, several researchers have investigated combined approaches between them or studied the effects of novel treatment options in association with classic antifungal drugs.

Karlsson et al. [135] have reported the fabrication of multilayered polyelectrolyte thin films (PEMs) that promote the surface-mediated release of an antifungal beta-peptide. Specifically, the researchers have incorporated a fluorescently labeled antifungal beta-peptide into the structures of PEMs fabricated from poly-L-glutamic acid and poly-L-lysine manufactured through a layer-by-layer process. The obtained materials showed promising ability in inhibiting the growth of *C. albicans* on film-coated surfaces.

Tonglairoum et al. [8] developed clotrimazole (CZ)-loaded microemulsion-containing nanofiber mats. They have successfully fabricated these mats by electrospinning a mixture of different CZ-microemulsion formulations polymer solutions. The researchers reported an initial burst release, followed by a sustained release of CZ. The mats presented remarkable antifungal properties, while the toxicity remained low. Nonetheless, it was concluded that further in vivo studies are required for material evaluation for the treatment of oral candidiasis.

Kong et al. [11] proposed a different approach by designing a bioadhesive hydroxypropyl methylcellulose hydrogel formulation of Histatin-5 for topical application against oral candidiasis. Histatin-5 was chosen due to its potency in killing *C. albicans*, without inducing resistance. The topical delivery through bioadhesive hydrogels is considered ideal as it provides extended release of the therapeutic agents, a desired characteristic for treating infections. Taking also into account the lack of toxicity, anti-inflammatory, and wound-healing properties of histatin-5, the findings of this study confirm the usefulness and commercial feasibility of this therapeutic strategy.

Another approach is offered by Nagrath et al. [149], who attempted to repurpose PMMA for 3D printing along with functionalization of the tissue surface using the controlled release of polycaprolactone (PCL) microspheres loaded with amphotericin B. The researchers obtained promising results as the 3D printed dentures presented comparable mechanical properties to conventionally fabricated ones, while the PCL-PMMA surface released the drug over sustained periods, actively reducing *C. albicans* colonization in a biomass assay.

Tejada et al. [26] mixed gelatin (GEL) and chitosan (CH) in various ratios to create natural polymeric blend-based nanoparticles aimed to deliver miconazole nitrate and lidocaine chlorohydrate. A faster release was observed when the GEL/CH ratio was higher, possibly due to GEL solubilization in the medium that led to the erosion of the polymer matrix and release of encapsulated drugs. Nanoparticle-encapsulation conducted to a sustained release for 24 h, indicating the potential of such systems to be included in a buccal film or a buccal tablet to obtain an alternative therapeutic formulation for the treatment of *C. albicans*.

## 5. Conclusions

To summarize, oral candidiasis can be a life-threatening infection for immunocompromised individuals, requiring strong antifungal drugs. The classic therapeutic approach implies the administration of different polyenes, azoles, or echinocandins, while prevention is ensured through good hygiene practices and attention to proper denture fit. To avoid *Candida* spp. overgrowth and limit the adverse effects associated to traditional antifungal agents, advances have been made for developing anti-*Candida* biomaterials.

By making use of inherently fungistatic or fungicidal polymeric, inorganic, and natural products, several strategies can be developed to prevent and fight these oral infections. Coating, functionalizing, and/or incorporating them into denture base materials are all considered efficient novel treatment options for oral candidiasis. Besides, delivering classic drugs via controlled delivery systems helps reducing adverse effect without hampering the therapeutic performance. Nonetheless, the alternatives combining several biomaterials approaches have been proven remarkably successful.

Therefore, the current and underdevelopment treatment options presented in this review can stand as inception points for further research. Considering the characteristics of each of the previously described compounds, biomaterials, and delivery methods, better oral hygiene products, prosthesis materials, denture adhesives, and therapeutic formulations can be created.

To conclude, there is an increased research interest towards developing innovative *Candida*-inhibiting biomaterials. However, despite the significant progress that has been made towards finding better oral candidiasis treatment strategies, there is still room for improvement. Particularly, most of the tested compounds and biomaterials have not yet advanced beyond preclinical testing, and special attention must also be given to currently understudied complex and mixed biofilms.

## Figures and Tables

**Table 1:** Comparison of in vitro susceptibilities of different *Candida* species to conventional antifungal agents. Adapted from [29], BMC Infectious Diseases, 2018.

	Antifungal Agent	Amphotericin			Fluconazole			Anidulafungin			Caspofungin		
*Candida* Species		MIC Range	MIC_50_	MIC_90_	MIC Range	MIC_50_	MIC_90_	MIC Range	MIC_50_	MIC_90_	MIC Range	MIC_50_	MIC_90_
*C. albicans*		0.016–16	1	4	0.063–64	0.5	8	0.008–0.25	0.031	0.125	0.008–8	0.25	1
*C. dubliniensis*		0.063–0.125	0.031	2	0.063–0.125	0.125	0.125	0.008–0.125	0.125	0.25	0.25–2	0.5	2
*C. glabrata*		0.016–4	1	2	0.25–64	8	64	0.016–1	0.063	1	0.008–2	0.5	2
*C. krusei*		0.063–2	0.5	1	0.25–64	8	64	0.016–0.25	0.125	0.25	0.063–4	2	4
*C. tropicalis*		0.031–2	1	2	0.063–8	4	8	0.008–0.063	0.063	0.063	0.031–8	0.5	8
*C. keyfr*		0.016–1	0.5	1	0.25–32	4	32	0.031–0.063	0.063	0.5	0.125–0.05	0.25	0.5

MIC—minimum inhibitory concentration (μg/mL).

**Table 2:** Antifungal medication.

Drug	Form	Dose	Indication	Adverse Effects	Refs.
Amphotericin B	Infusion	100–200 mg/6 h	Intraoral candidiasis, chronic erythematous candidiasis	Renal, cardiovascular, spinal and neurological effects	[10,36,37]
Nystatin	Suspension	4–6 mL/6 h	Intraoral candidiasis	Well tolerated	[10,36]
	Ointment	2–4 applications/day	Angular cheilitis	Well tolerated	[10,36]
	Tablets/Pastilles	2 every 8 h	Denture stomatitis	Uncommon effects: nausea, vomiting, gastrointestinal effects	[36,38]
Fluconazole	Tablets	50–100 mg/day	Pseudomembranous candidiasis, acute erythematous candidiasis, chronic hyperplastic candidiasis	Nausea, vomiting, diarrhea, abdominal pain	[36,37]
	Suspension	10 mg/mL	Oropharyngeal candidiasis	Nausea, vomiting, diarrhea, abdominal pain	[36,37,39]
Miconazole	Gel/cream	100 mg/6 h	Angular cheilitis, chronic erythematous candidiasis	Uncommon effects: burning, irritation, nausea, diarrhea	[10,36,37]
Ketoconazole	Gel/cream	3 times/day	Angular cheilitis	Nausea, vomiting	[10,36]
	Tablets	200 mg, 2–2/day	Pseudomembranous candidiasis, acute erythematous candidiasis, chronic hyperplastic candidiasis	Abdominal pain	[36,37]
Clotrimazole	Gel/cream	3 times/day	Angular cheilitis	Occasional effects: skin irritation, burning sensation	[10,36]
	Tablets/troches	5 times/day	Intraoral candidiasis	Occasional effects: skin irritation, burning sensation	[10,36]
Betamethasone dipropionate clotrimazole	Cream	4 times/day	Chronic angular cheilitis	Local irritation	[10,40,41,42]
Itraconazole	Capsules	100–200 mg/day	Pseudomembranous candidiasis, acute erythematous candidiasis, chronic hyperplastic candidiasis	Nausea, vomiting, diarrhea, abdominal pain	[36,37]
Voriconazole	Infusion	First day: 6 mg/kg once every 12 h Rest of the treatment: 4 mg/kg once every 12 h	Intraoral candidiasis	Neuropsychiatric and gastrointestinal effects	[37,40,43,44]
	Tablets	First day: 200–400 mg once every 12 h Rest of the treatment: 100–200 mg once every 12 h	Intraoral candidiasis	Neuropsychiatric and gastrointestinal effects	[37,40,43,44]
Posaconazole	Oral suspension/Tablets	First week: 200 mg, 4 times/day Rest of the treatment: 400 mg, 2 times/day	Oropharyngeal candidiasis	Headaches, gastrointestinal effects	[37,40,45]
Anidulafungin	Infusion	First day: 3 mg/kg/day (max 200 mg) Rest of the treatment: 1.5 mg/kg/day (max 100 mg)	Invasive candidiasis	Occasional effects: anemia, diarrhea, pyrexia, vomiting, hypokalemia	[19,46,47]
Caspofungin	Infusion	First day: 70 mg/day Rest of the treatment: 50 mg/day	Invasive candidiasis	Occasional effects: phlebitis, fever, abdominal pain, nausea, diarrhea, headache, rash, leukopenia, hypokalemia	[19,47]
Micafungin	Infusion	1–2 mg/kg/day (max 100 mg/day)	Invasive candidiasis	Occasional effects: fever, nausea, headache, rash	[19,47]

## Data Availability

Not applicable.

## References

[1] Arweiler N.B., Netuschil L., Schwiertz A. (2016). The Oral Microbiota. Microbiota of the Human Body: Implications in Health and Disease.

[2] Coll P.P., Lindsay A., Meng J., Gopalakrishna A., Raghavendra S., Bysani P., O’Brien D. (2020). The Prevention of Infections in Older Adults: Oral Health. J. Am. Geriatr. Soc..

[3] Lee J., Kim J.-G., Lee H., Lee T.H., Kim K.-Y., Kim H. (2021). Antifungal Activity of 1,4-Dialkoxynaphthalen-2-Acyl Imidazolium Salts by Inducing Apoptosis of Pathogenic *Candida* spp.. Pharmaceutics.

[4] Volkova M., Atamas A., Tsarenko A., Rogachev A., Guskov A. (2021). Cation Transporters of *Candida albicans*—New Targets to Fight Candidiasis?. Biomolecules.

[5] Singh A., Verma R., Murari A., Agrawal A. (2014). Oral candidiasis: An overview. J. Oral Maxillofac. Pathol..

[6] Sharon V., Fazel N. (2010). Oral candidiasis and angular cheilitis. Dermatol. Ther..

[7] Muñoz J.E., Rossi D.C.P., Jabes D.L., Barbosa D.A., Cunha F.F.M., Nunes L.R., Arruda D.C., Pelleschi Taborda C. (2020). In Vitro and In Vivo Inhibitory Activity of Limonene against Different Isolates of *Candida* spp.. J. Fungi.

[8] Tonglairoum P., Ngawhirunpat T., Rojanarata T., Kaomongkolgit R., Opanasopit P. (2015). Fabrication of a novel scaffold of clotrimazole-microemulsion-containing nanofibers using an electrospinning process for oral candidiasis applications. Colloids Surf. B Biointerfaces.

[9] Siqueira A.B., Rodriguez L.R., Santos R.K., Marinho R.R., Abreu S., Peixoto R.F., Gurgel B.C. (2015). Antifungal activity of propolis against *Candida* species isolated from cases of chronic periodontitis. Braz. Oral Res..

[10] Dangi Y.S., Soni M.L., Namdeo K.P. (2010). Oral candidiasis: A review. Int. J. Pharm. Pharm. Sci..

[11] Kong E.F., Tsui C., Boyce H., Ibrahim A., Hoag S.W., Karlsson A.J., Meiller T.F., Jabra-Rizk M.A. (2016). Development and In Vivo Evaluation of a Novel Histatin-5 Bioadhesive Hydrogel Formulation against Oral Candidiasis. Antimicrob. Agents Chemother..

[12] Arastehfar A., Carvalho A., Nguyen M.H., Hedayati M.T., Netea M.G., Perlin D.S., Hoenigl M. (2020). COVID-19-Associated Candidiasis (CAC): An Underestimated Complication in the Absence of Immunological Predispositions?. J. Fungi.

[13] Dutta T., Ghosh N.N., Das M., Adhikary R., Mandal V., Chattopadhyay A.P. (2020). Green synthesis of antibacterial and antifungal silver nanoparticles using *Citrus limetta* peel extract: Experimental and theoretical studies. J. Environ. Chem. Eng..

[14] Muadcheingka T., Tantivitayakul P. (2015). Distribution of *Candida albicans* and non-albicans *Candida* species in oral candidiasis patients: Correlation between cell surface hydrophobicity and biofilm forming activities. Arch. Oral Biol..

[15] Khan A.A., Alanazi A.M., Alsaif N., Algrain N., Wani T.A., Bhat M.A. (2021). Enhanced Efficacy of Thiosemicarbazone Derivative-Encapsulated Fibrin Liposomes against Candidiasis in Murine Model. Pharmaceutics.

[16] Ficanha A.M.M., Antunes A., Oro C.E.D., Dallago R.M., Mignoni M.L. (2020). Immobilization of *Candida antarctica* B (CALB) in Silica Aerogel: Morphological Characteristics and Stability. Biointerface Res. Appl. Chem..

[17] Wiench R., Skaba D., Matys J., Grzech-Leśniak K. (2021). Efficacy of Toluidine Blue—Mediated Antimicrobial Photodynamic Therapy on *Candida* spp. A Systematic Review. Antibiotics.

[18] Roozbehani N., Golfeshan F., Pakshir K., Doorandishan M., Jassbi A.R., Mosaddad S.A. (2021). Chemical Composition and Effectiveness of *Ocimum basilicum* L. Extracts on the Adhesion of *Candida albicans* and *C. dubliniensis* on Acrylic Surfaces of Removable Orthodontic Appliances. Biointerface Res. Appl. Chem..

[19] Quindós G., Gil-Alonso S., Marcos-Arias C., Sevillano E., Mateo E., Jauregizar N., Eraso E. (2019). Therapeutic tools for oral candidiasis: Current and new antifungal drugs. Med. Oral Patol. Oral Cir. Bucal.

[20] Jung J., Li L., Yeh C.-K., Ren X., Sun Y. (2019). Amphiphilic quaternary ammonium chitosan/sodium alginate multilayer coatings kill fungal cells and inhibit fungal biofilm on dental biomaterials. Mater. Sci. Eng. C.

[21] Vera-González N., Shukla A. (2020). Advances in Biomaterials for the Prevention and Disruption of Candida Biofilms. Front. Microbiol..

[22] Namangkalakul W., Benjavongkulchai S., Pochana T., Promchai A., Satitviboon W., Howattanapanich S., Phuprasong R., Ungvijanpunya N., Supakanjanakanti D., Chaitrakoonthong T. (2020). Activity of chitosan antifungal denture adhesive against common *Candida* species and *Candida albicans* adherence on denture base acrylic resin. J. Prosthet. Dent..

[23] Okonogi S., Phumat P., Khongkhunthian S., Suttiat K., Chaijareenont P. (2021). Denture-Soaking Solution Containing Piper betle Extract-Loaded Polymeric Micelles; Inhibition of *Candida albicans*, Clinical Study, and Effects on Denture Base Resin. Antibiotics.

[24] Lamfon H.A. (2021). Denture Biofilm and Dentureassociated Stomatitis, A Literature Review. Egypt. Dent. J..

[25] Takamiya A.S., Monteiro D.R., Gorup L.F., Silva E.A., de Camargo E.R., Gomes-Filho J.E., de Oliveira S.H.P., Barbosa D.B. (2021). Biocompatible silver nanoparticles incorporated in acrylic resin for dental application inhibit *Candida albicans* biofilm. Mater. Sci. Eng. C.

[26] Tejada G., Barrera M.G., García P., Sortino M., Lamas M.C., Lassalle V., Alvarez V., Leonardi D. (2020). Nanoparticulated Systems Based on Natural Polymers Loaded with Miconazole Nitrate and Lidocaine for the Treatment of Topical Candidiasis. AAPS PharmSciTech.

[27] Cuéllar-Cruz M., Vega-González A., Mendoza-Novelo B., López-Romero E., Ruiz-Baca E., Quintanar-Escorza M.A., Villagómez-Castro J.C. (2012). The effect of biomaterials and antifungals on biofilm formation by *Candida* species: A review. Eur. J. Clin. Microbiol. Infect. Dis..

[28] Muhvić-Urek M., Saltović E., Braut A., Kovačević Pavičić D. (2020). Association between Vitamin D and Candida-Associated Denture Stomatitis. Dent. J..

[29] Aslani N., Janbabaei G., Abastabar M., Meis J.F., Babaeian M., Khodavaisy S., Boekhout T., Badali H. (2018). Identification of uncommon oral yeasts from cancer patients by MALDI-TOF mass spectrometry. BMC Infect. Dis..

[30] Coronado-Castellote L., Jiménez-Soriano Y. (2013). Clinical and microbiological diagnosis of oral candidiasis. J. Clin. Exp. Dent..

[31] Colombo A.L., de Almeida Júnior J.N., Slavin M.A., Chen S.C.A., Sorrell T.C. (2017). Candida and invasive mould diseases in non-neutropenic critically ill patients and patients with haematological cancer. Lancet Infect. Dis..

[32] Reichart P.A., Samaranayake L.P., Philipsen H.P. (2000). Pathology and clinical correlates in oral candidiasis and its variants: A review. Oral Dis..

[33] Araujo V.H.S., Duarte J.L., Carvalho G.C., Silvestre A.L.P., Fonseca-Santos B., Marena G.D., Ribeiro T.D.C., dos Santos Ramos M.A., Bauab T.M., Chorilli M. (2020). Nanosystems against candidiasis: A review of studies performed over the last two decades. Crit. Rev. Microbiol..

[34] Abraham C.M. (2011). Advances and emerging techniques in the identification, diagnosis and treatment of oral candidiasis. Open Pathol. J..

[35] Kofla G., Ruhnke M. (2011). Pharmacology and metabolism of anidulafungin, caspofungin and micafungin in the treatment of invasive candidosis—Review of the literature. Eur. J. Med. Res..

[36] Garcia-Cuesta C., Sarrion-Pérez M.-G., Bagán J.V. (2014). Current treatment of oral candidiasis: A literature review. J. Clin. Exp. Dent..

[37] Williams D., Lewis M. (2011). Pathogenesis and treatment of oral candidosis. J. Oral Microbiol..

[38] Lyu X., Zhao C., Yan Z.-M., Hua H. (2016). Efficacy of nystatin for the treatment of oral candidiasis: A systematic review and meta-analysis. Drug Des. Dev. Ther..

[39] Govindarajan A., Bistas K.G., Aboeed A. (2020). Fluconazole.

[40] Manik A., Bahl R. (2017). A review on oral candidal infection. J. Adv. Med. Dent. Sci. Res..

[41] Vigneswaran N., Muller S., Jeske A.H. (2019). Pharmacologic Management of Oral Mucosal Inflammatory and Ulcerative Diseases. Contemporary Dental Pharmacology: Evidence-Based Considerations.

[42] Hengge U.R., Ruzicka T., Schwartz R.A., Cork M.J. (2006). Adverse effects of topical glucocorticosteroids. J. Am. Acad. Dermatol..

[43] Levêque D., Nivoix Y., Jehl F., Herbrecht R. (2006). Clinical pharmacokinetics of voriconazole. Int. J. Antimicrob. Agents.

[44] Levine M.T., Chandrasekar P.H. (2016). Adverse effects of voriconazole: Over a decade of use. Clin. Transplant..

[45] Torres H.A., Hachem R.Y., Chemaly R.F., Kontoyiannis D.P., Raad I.I. (2005). Posaconazole: A broad-spectrum triazole antifungal. Lancet Infect. Dis..

[46] Roilides E., Carlesse F., Tawadrous M., Leister-Tebbe H., Conte U., Raber S., Swanson R., Yan J.L., Aram J.A., Queiroz-Telles F. (2020). Safety, Efficacy and Pharmacokinetics of Anidulafungin in Patients 1 Month to <2 Years of Age with Invasive Candidiasis, Including Candidemia. Pediatr. Infect. Dis. J..

[47] Glöckner A. (2011). Treatment and prophylaxis of invasive candidiasis with anidulafungin, caspofungin and micafungin—Review of the literature. Eur. J. Med. Res..

[48] Ahmad N., Jafri Z., Khan Z.H. (2020). Evaluation of nanomaterials to prevent oral Candidiasis in PMMA based denture wearing patients. A systematic analysis. J. Oral Biol. Craniofac. Res..

[49] Antunes D.P., Salvia A.C.R.D., de Araújo R.M., Di Nicoló R., Koga Ito C.Y., de Araujo M.A.M. (2015). Effect of green tea extract and mouthwash without alcohol on *Candida albicans* biofilm on acrylic resin. Gerodontology.

[50] Paulone S., Malavasi G., Ardizzoni A., Orsi C.F., Peppoloni S., Neglia R.G., Blasi E. (2017). *Candida albicans* survival, growth and biofilm formation are differently affected by mouthwashes: An in vitro study. New Microbiol..

[51] Vila T., Sultan A.S., Montelongo-Jauregui D., Jabra-Rizk M.A. (2020). Oral Candidiasis: A Disease of Opportunity. J. Fungi.

[52] Spirescu V.A., Chircov C., Grumezescu A.M., Andronescu E. (2021). Polymeric Nanoparticles for Antimicrobial Therapies: An up-to-date Overview. Polymers.

[53] Cheraghipour K., Ezatpour B., Masoori L., Marzban A., Sepahvand A., Rouzbahani A.K., Moridnia A., Khanizadeh S., Mahmoudvand H. (2021). Anti-Candida activity of Curcumin: A systematic review. Curr. Drug Discov. Technol..

[54] Meireles A.B., Corrêa D.K., da Silveira J.V.W., Millás A.L.G., Bittencourt E., de Brito-Melo G.E.A., González-Torres L.A. (2018). Trends in polymeric electrospun fibers and their use as oral biomaterials. Exp. Biol. Med..

[55] Fakhri E., Eslami H., Maroufi P., Pakdel F., Taghizadeh S., Ganbarov K., Yousefi M., Tanomand A., Yousefi B., Mahmoudi S. (2020). Chitosan biomaterials application in dentistry. Int. J. Biol. Macromol..

[56] Malviya R. (2020). Exploration of neem gum-chitosan and kheri gum-chitosan polyelectrolyte complex based film for transdermal delivery of protein/peptide. Biointerface Res. Appl. Chem..

[57] Tajdini K., Shakeri A., Naijian F. (2020). Nanocomposite hydrogel of chitosan-g-poly acrylamide/nanoclay: Effect of degree of cross-linking on their swelling. Lett. Appl. NanoBioSci..

[58] Srimaneepong V., Thanamee T., Wattanasirmkit K., Muangsawat S., Matangkasombut O. (2021). Efficacy of low-molecular weight chitosan against *Candida albicans* biofilm on polymethyl methacrylate resin. Aust. Dent. J..

[59] Ikono R., Vibriani A., Wibowo I., Saputro K.E., Muliawan W., Bachtiar B.M., Mardliyati E., Bachtiar E.W., Rochman N.T., Kagami H. (2019). Nanochitosan antimicrobial activity against *Streptococcus mutans* and *Candida albicans* dual-species biofilms. BMC Res. Notes.

[60] Liu R., Chen X., Falk S.P., Mowery B.P., Karlsson A.J., Weisblum B., Palecek S.P., Masters K.S., Gellman S.H. (2014). Structure–Activity Relationships among Antifungal Nylon-3 Polymers: Identification of Materials Active against Drug-Resistant Strains of *Candida albicans*. J. Am. Chem. Soc..

[61] Velazco-Medel M.A., Camacho-Cruz L.A., Lugo-Gonzalez J.C., Bucio E. (2020). Antifungal polymers for medical applications. Med. Devices Sens..

[62] Rank L.A., Walsh N.M., Liu R., Lim F.Y., Bok J.W., Huang M., Keller N.P., Gellman S.H., Hull C.M. (2017). A Cationic Polymer That Shows High Antifungal Activity against Diverse Human Pathogens. Antimicrob. Agents Chemother..

[63] Rank L.A., Walsh N.M., Lim F.Y., Gellman S.H., Keller N.P., Hull C.M. (2018). Peptide-like nylon-3 polymers with activity against phylogenetically diverse, intrinsically drug-resistant pathogenic fungi. mSphere.

[64] Martini Garcia I., Becker Rodrigues S., Rodrigues Gama M.E., Branco Leitune V.C., Melo M.A., Mezzomo Collares F. (2020). Guanidine derivative inhibits *C. albicans* biofilm growth on denture liner without promote loss of materials’ resistance. Bioact. Mater..

[65] Dias F.G.G., Pereira L.d.F., Parreira R.L.T., Veneziani R.C.S., Bianchi T.C., Fontes V.F.N.D.P., Galvani M.D.C., Cerce D.D.P., Martins C.H.G., Rinaldi-Neto F. (2021). Evaluation of the antiseptic and wound healing potential of polyhexamethylene guanidine hydrochloride as well as its toxic effects. Eur. J. Pharm. Sci..

[66] Gama M.E.R., Leitune V.C.B., Garcia I.M., Rodrigues S.B., Collares F.M. (2020). Evaluation of guanidine antifungal solutions for denture base resin: An in vitro study. Rev. Fac. Odontol. Porto Alegre.

[67] Choi H., Kim K.-J., Lee D.G. (2017). Antifungal activity of the cationic antimicrobial polymer-polyhexamethylene guanidine hydrochloride and its mode of action. Fungal Biol..

[68] Husain Q. (2019). An overview on the green synthesis of nanoparticles and other nano-materials using enzymes and their potential applications. Biointerface Res. Appl. Chem..

[69] Ratna Geetika G., Raji P., Bennet Rohan D., Divya Kumar M., Kripu Sharma V., Keerthana D., Karishma S., Iyappan P., Thirumurugan R., Samrot A.V. (2020). Green synthesis and antibacterial activity of silver nanoparticles from the aqueous extracts of *Cassia alata*. Lett. Appl. NanoBioSci..

[70] Gherasim O., Puiu R.A., Bîrcă A.C., Burdușel A.-C., Grumezescu A.M. (2020). An Updated Review on Silver Nanoparticles in Biomedicine. Nanomaterials.

[71] Burdușel A.-C., Gherasim O., Grumezescu A.M., Mogoantă L., Ficai A., Andronescu E. (2018). Biomedical Applications of Silver Nanoparticles: An Up-to-Date Overview. Nanomaterials.

[72] Fajar M.N., Endarko E., Rubiyanto A., Malek N., Hadibarata T., Syafiuddin A. (2019). A green deposition method of silver nanoparticles on textiles and their antifungal activity. Biointerface Res. Appl. Chem..

[73] Gupta K., Chundawat T.S., Malek N.A.N.N. (2020). Antibacterial, Antifungal, Photocatalytic Activities and Seed Germination Effect of Mycosynthesized Silver Nanoparticles using *Fusarium oxysporum*. Biointerface Res. Appl. Chem..

[74] Ratnasari A., Endarko E., Syafiuddin A. (2020). A Green Method for the Enhancement of Antifungal Properties of Various Textiles Functionalized with Silver Nanoparticles. Biointerface Res. Appl. Chem..

[75] Thiruvengadam V., Bansod A.V. (2020). Characterization of Silver Nanoparticles Synthesized using Chemical Method and its Antibacterial Property. Biointerface Res. Appl. Chem..

[76] Geetanjali, Sharma P.K., Malviya R. (2020). Toxicity and application of nano-silver in multi-drug resistant therapy. Lett. Appl. NanoBioSci..

[77] Pathak J., Sonker A.S., Rajneesh V.S., Kumar D., Sinha R.P. (2019). Synthesis of silver nanoparticles from extracts of *Scytonema geitleri* HKAR-12 and their in vitro antibacterial and antitumor potentials. Lett. Appl. NanoBioSci..

[78] Xue B., He D., Gao S., Wang D., Yokoyama K., Wang L. (2016). Biosynthesis of silver nanoparticles by the fungus *Arthroderma fulvum* and its antifungal activity against genera of *Candida*, *Aspergillus* and *Fusarium*. Int. J. Nanomed..

[79] Tyagi P.K., Mishra R., Khan F., Gupta D., Gola D. (2020). Antifungal Effects of Silver Nanoparticles Against Various Plant Pathogenic Fungi and its Safety Evaluation on *Drosophila melanogaster*. Biointerface Res. Appl. Chem..

[80] Hashim T., Risan M.H., Kadhom M., Raheem R., Yousif E. (2020). Antifungal, Antiviral, and Antibacterial Activities of Silver Nanoparticles Synthesized Using Fungi: A Review. Lett. Appl. NanoBioSci..

[81] Monteiro D.R., Gorup L.F., Silva S., Negri M., de Camargo E.R., Oliveira R., Barbosa D.B., Henriques M. (2011). Silver colloidal nanoparticles: Antifungal effect against adhered cells and biofilms of *Candida albicans* and *Candida glabrata*. Biofouling.

[82] Kanchi S., Inamuddin, Khan A. (2020). Biogenic Synthesis of Selenium Nanoparticles with Edible Mushroom Extract: Evaluation of Cytotoxicity on Prostate Cancer Cell Lines and Their Antioxidant, and Antibacterial Activity. Biointerface Res. Appl. Chem..

[83] Shakibaie M., Mohazab N.S., Mousavi S.A.A. (2015). Antifungal Activity of Selenium Nanoparticles Synthesized by *Bacillus* species Msh-1 Against *Aspergillus fumigatus* and *Candida albicans*. Jundishapur J. Microbiol..

[84] Guisbiers G., Lara H., Mendoza-Cruz R., Naranjo G., Vincent B.A., Peralta X.G., Nash K.L. (2017). Inhibition of *Candida albicans* biofilm by pure selenium nanoparticles synthesized by pulsed laser ablation in liquids. Nanomedicine.

[85] Parveen S., Wani A.H., Shah M.A., Devi H.S., Bhat M.Y., Koka J.A. (2018). Preparation, characterization and antifungal activity of iron oxide nanoparticles. Microb. Pathog..

[86] Seddighi N.S., Salari S., Izadi A.R. (2017). Evaluation of antifungal effect of iron-oxide nanoparticles against different *Candida* species. IET Nanobiotechnol..

[87] Sangaiya P., Jayaprakash R. (2018). A Review on Iron Oxide Nanoparticles and Their Biomedical Applications. J. Supercond. Nov. Magn..

[88] Samrot A.V., Sahithya C.S., Sruthi P.D., Selvarani A.J., Raji P., Prakash P., Ponnaiah P., Petchi I., Pattammadath S., Purayil S.K. (2020). Itraconazole Coated Super Paramagnetic Iron Oxide Nanoparticles for Antimicrobial Studies. Biointerface Res. Appl. Chem..

[89] Lipovsky A., Nitzan Y., Gedanken A., Lubart R. (2011). Antifungal activity of ZnO nanoparticles—The role of ROS mediated cell injury. Nanotechnology.

[90] Pillai A.M., Sivasankarapillai V.S., Rahdar A., Joseph J., Sadeghfar F., Anuf A R., Rajesh K., Kyzas G.Z. (2020). Green synthesis and characterization of zinc oxide nanoparticles with antibacterial and antifungal activity. J. Mol. Struct..

[91] Jamdagni P., Khatri P., Rana J.S. (2018). Green synthesis of zinc oxide nanoparticles using flower extract of *Nyctanthes arbor-tristis* and their antifungal activity. J. King Saud Univ. Sci..

[92] Souza J.M.T., de Araújo A.R., de Carvalho A.M.A., Amorim A.D.G.N., Daboit T.C., de Almeida J.R.D.S., da Silva D.A., Eaton P. (2020). Sustainably produced cashew gum-capped zinc oxide nanoparticles show antifungal activity against *Candida parapsilosis*. J. Clean. Prod..

[93] Spirescu V.A., Chircov C., Grumezescu A.M., Vasile B.Ș., Andronescu E. (2021). Inorganic Nanoparticles and Composite Films for Antimicrobial Therapies. Int. J. Mol. Sci..

[94] Sawai J., Yoshikawa T. (2004). Quantitative evaluation of antifungal activity of metallic oxide powders (MgO, CaO and ZnO) by an indirect conductimetric assay. J. Appl. Microbiol..

[95] Kong F., Wang J., Han R., Ji S., Yue J., Wang Y., Ma L. (2020). Antifungal Activity of Magnesium Oxide Nanoparticles: Effect on the Growth and Key Virulence Factors of *Candida albicans*. Mycopathologia.

[96] Amrulloh H., Fatiqin A., Simanjuntak W., Afriyani H., Annissa A. (2021). Bioactivities of nano-scale magnesium oxide prepared using aqueous extract of *Moringa oleifera* leaves as green agent. Adv. Nat. Sci. Nanosci. Nanotechnol..

[97] Kamboj A., Amjad M., Ahmad W., Singh A. (2020). A general survey on Green synthesis and application of calcium oxide nanoparticles. Int. J. Health Clin. Res..

[98] Amiri M., Etemadifar Z., Daneshkazemi A., Nateghi M. (2017). Antimicrobial Effect of Copper Oxide Nanoparticles on Some Oral Bacteria and *Candida* Species. J. Dent. Biomater..

[99] Imani M.M., Safaei M., Moradpoor H., Rezaei R., Golshah A., Rezaei F., Jamshidy L. (2020). Optimum synthesis of CuO nanoparticles with the highest antifungal activity against oral pathogen *Candida albicans*. J. Appl. Pharm. Sci..

[100] Padmavathi A.R., Murthy S.P., Das A., Priya A., Sushmitha T.J., Pandian S.K., Toleti S.R. (2020). Impediment to growth and yeast-to-hyphae transition in *Candida albicans* by copper oxide nanoparticles. Biofouling.

[101] Dizaj S.M., Lotfipour F., Barzegar-Jalali M., Zarrintan M.H., Adibkia K. (2014). Antimicrobial activity of the metals and metal oxide nanoparticles. Mater. Sci. Eng. C.

[102] Haghighi F., Mohammadi S.R., Mohammadi P., Eskandari M., Hosseinkhani S. (2012). The evaluation of *Candida albicans* biofilms formation on silicone catheter, PVC and glass coated with titanium dioxide nanoparticles by XTT method and ATPase assay. Bratisl. Lek. Listy.

[103] Ahmad N.S., Abdullah N., Yasin F.M. (2019). Antifungal activity of titanium dioxide nanoparticles against *Candida albicans*. BioResources.

[104] Haghighi F., Roudbar Mohammadi S., Mohammadi P., Hosseinkhani S., Shipour R. (2013). Antifungal activity of TiO_2_ nanoparticles and EDTA on *Candida albicans* biofilms. Infect. Epidemiol. Microbiol..

[105] Hernandez-Delgadillo R., Velasco-Arias D., Martinez-Sanmiguel J.J., Diaz D., Zumeta-Dube I., Arevalo-Niño K., Cabral-Romero C. (2013). Bismuth oxide aqueous colloidal nanoparticles inhibit *Candida albicans* growth and biofilm formation. Int. J. Nanomed..

[106] El-Batal A.I., El-Sayyad G.S., El-Ghamry A., Agaypi K.M., Elsayed M.A., Gobara M. (2017). Melanin-gamma rays assistants for bismuth oxide nanoparticles synthesis at room temperature for enhancing antimicrobial, and photocatalytic activity. J. Photochem. Photobiol. B Biol..

[107] Haq S., Rehman W., Waseem M., Meynen V., Awan S.U., Saeed S., Iqbal N. (2018). Fabrication of pure and moxifloxacin functionalized silver oxide nanoparticles for photocatalytic and antimicrobial activity. J. Photochem. Photobiol. B Biol..

[108] Mohammed H.B., Rayyif S.M.I., Curutiu C., Birca A.C., Oprea O.-C., Grumezescu A.M., Ditu L.-M., Gheorghe I., Chifiriuc M.C., Mihaescu G. (2021). Eugenol-Functionalized Magnetite Nanoparticles Modulate Virulence and Persistence in *Pseudomonas aeruginosa* Clinical Strains. Molecules.

[109] Holban A.M., Grumezescu A.M., Ficai A., Chifiriuc C.M., Lazar V., Radulescu R. (2013). Fe_3_O_4_@C18-Carvone to Prevent *Candida Tropicalis* Biofilm Development. Rev. Romana Mater..

[110] Nuță D.C., Limban C., Chiriță C., Chifiriuc M.C., Costea T., Ioniță P., Nicolau I., Zarafu I. (2021). Contribution of Essential Oils to the Fight against Microbial Biofilms—A Review. Processes.

[111] Miao Q., Zhao L., Wang Y., Hao F., Sun P., He P., Liu Y., Huang J., Liu X., Liu X. (2020). Microbial metabolomics and network analysis reveal fungistatic effect of basil (*Ocimum basilicum*) oil on *Candida albicans*. J. Ethnopharmacol..

[112] Waskito H., Apriasari M.L., Utami J.P. (2020). Antifungal Effect of Mauli Banana Stem Extract, Basil Leaf Extract, And their Combination on *Candida albicans*. Dent. J. Kedokt. Gigi.

[113] Sugio C.Y.C., Mengoa M.G.R., Gomes A.C.G., Garcia A.A.M.N., de Oliveira T.M., Hermana K. (2020). Use of Natural Products in the Prevention and Treatment of Denture Stomatitis. Open Access J. Biomed. Sci..

[114] Masłowski M., Miedzianowska J., Czylkowska A., Strzelec K. (2020). Horsetail (*Equisetum arvense*) as a Functional Filler for Natural Rubber Biocomposites. Materials.

[115] Briceño-Cardona K.L., Romero C.C., Delgadillo R.H., Galindo-Rodríguez S.A., Solís-Soto J.M. (2021). Equisetum extracts are anti-inflammatory and antibacterial, an oral potential therapeutic agent. Int. J. Appl. Dent. Sci..

[116] Martins Almeida N.L., Saldanha L.L., Alves da Silva R., Pinke K.H., da Costa E.F., Porto V.C., Dokkedal A.L., Soares Lara V. (2018). Antimicrobial activity of denture adhesive associated with *Equisetum giganteum*-and *Punica granatum*-enriched fractions against *Candida albicans* biofilms on acrylic resin surfaces. Biofouling.

[117] Trifan A., Bostănaru A.-C., Luca S.V., Grădinaru A.C., Jităreanu A., Aprotosoaie A.C., Miron A., Cioancă O., Hăncianu M., Ochiuz L. (2020). Antifungal potential of *Pimpinella anisum*, *Carum carvi* and *Coriandrum sativum* extracts. A comparative study with focus on the phenolic composition. Farmacia.

[118] Silva F., Domeño C., Domingues F.C., Preedy V.R., Watson R.R. (2020). Chapter 35—*Coriandrum sativum* L.: Characterization, Biological Activities, and Applications. Nuts and Seeds in Health and Disease Prevention.

[119] Furletti V.F., Teixeira I.P., Obando-Pereda G., Mardegan R.C., Sartoratto A., Figueira G.M., Duarte R.M.T., Rehder V.L.G., Duarte M.C.T., Höfling J.F. (2011). Action of *Coriandrum sativum* L. Essential Oil upon Oral *Candida albicans* Biofilm Formation. Evid. Based Complement. Altern. Med..

[120] Ashyuce S., Bereli N., Topcu A., Ramteke P.W., Denizli A. (2019). Indian saffron—Turmeric (*Curcuma longa*) embedded supermacroporous cryogel discs for heavy metal removal. Biointerface Res. Appl. Chem..

[121] Costa Normando A.G., Gomes de Meneses A., Porto de Toledo I., Alvares Borges G., Lourenco de Lima C., Diniz Dos Reis P.E., Silva Guerra E.N. (2019). Effects of turmeric and curcumin on oral mucositis: A systematic review. Phytother. Res..

[122] Adamczak A., Ożarowski M., Karpiński T.M. (2020). Curcumin, a Natural Antimicrobial Agent with Strain-Specific Activity. Pharmaceuticals.

[123] Raduly F.M., Raditoiu V., Raditoiu A., Purcar V. (2021). Curcumin: Modern Applications for a Versatile Additive. Coatings.

[124] Mohammadian M., Moghaddam A.D., Sharifan A., Dabaghi P., Hadi S. (2020). Different forms of whey protein aggregates as curcumin delivery systems: Evaluation of free radical scavenging activity and drug release kinetics. Biointerface Res. Appl. Chem..

[125] Narayanan V.S., Muddaiah S., Shashidara R., Sudheendra U.S., Deepthi N.C., Samaranayake L. (2020). Variable antifungal activity of curcumin against planktonic and biofilm phase of different candida species. Indian J. Dent. Res..

[126] Soto-Chilaca G.A., Mejia-Garibay B., Navarro-Amador R., Ramirez-Corona N., Palou E., Lopez-Malo A. (2019). Cinnamaldehyde-loaded chitosan nanoparticles: Characterization and antimicrobial activity. Biointerface Res. Appl. Chem..

[127] Yanakiev S. (2020). Effects of Cinnamon (*Cinnamomum* spp.) in Dentistry: A Review. Molecules.

[128] Ranasinghe P., Pigera S., Premakumara G.A.S., Galappaththy P., Constantine G.R., Katulanda P. (2013). Medicinal properties of ‘true’ cinnamon (*Cinnamomum zeylanicum*): A systematic review. BMC Complement. Altern. Med..

[129] Jafri H., Ansari F.A., Ahmad I., Ahmad Khan M.S., Ahmad I., Chattopadhyay D. (2019). Chapter 9—Prospects of Essential Oils in Controlling Pathogenic Biofilm. New Look to Phytomedicine.

[130] Shahidi Noghabi M., Molaveisi M. (2020). The effect of wall formulation on storage stability and physicochemical properties of cinnamon essential oil microencapsulated by spray drying. Chem. Pap..

[131] De Araújo M.R.C., Maciel P.P., Castellano L.R.C., Bonan P.R.F., Alves D.D.N., de Medeiros A.C.D., de Castro R.D. (2021). Efficacy of essential oil of cinnamon for the treatment of oral candidiasis: A randomized trial. Spec. Care Dent..

[132] Ota C., Unterkircher C., Fantinato V., Shimizu M.T. (2001). Antifungal activity of propolis on different species of *Candida*. Mycoses.

[133] Kumar D., Ayesha M.J., Gautam P., Joshi H., Kumar N. (2020). A Recent Report on ‘Plants with Anti-Candida Properties’. Int. J. Curr. Res. Rev..

[134] De Oliveira S.G.D., Martos J., de Carvalho R.V., de Pereira C.M.P., Lund R.G., Piva E. (2021). Retentive efficacy, antimicrobial and cytotoxicity comparisons between different types of commercial and experimental denture adhesives with antifungal action. Dent. Mater. J..

[135] Karlsson A.J., Flessner R.M., Gellman S.H., Lynn D.M., Palecek S.P. (2010). Polyelectrolyte multilayers fabricated from antifungal β-peptides: Design of surfaces that exhibit antifungal activity against *Candida albicans*. Biomacromolecules.

[136] Ramasamy M., Lee J. (2016). Recent Nanotechnology Approaches for Prevention and Treatment of Biofilm-Associated Infections on Medical Devices. BioMed Res. Int..

[137] Ali Sabri B., Satgunam M., Abreeza N.M., Abed A.N. (2021). A review on enhancements of PMMA Denture Base Material with Different Nano-Fillers. Cogent Eng..

[138] Cierech M., Kolenda A., Grudniak A.M., Wojnarowicz J., Woźniak B., Gołaś M., Swoboda-Kopeć E., Łojkowski W., Mierzwińska-Nastalska E. (2016). Significance of polymethylmethacrylate (PMMA) modification by zinc oxide nanoparticles for fungal biofilm formation. Int. J. Pharm..

[139] Mangal U., Kim J.-Y., Seo J.-Y., Kwon J.-S., Choi S.-H. (2019). Novel Poly (Methyl Methacrylate) Containing Nanodiamond to Improve the Mechanical Properties and Fungal Resistance. Materials.

[140] Acosta-Torres L.S., Mendieta I., Nuñez-Anita R.E., Cajero-Juárez M., Castaño V.M. (2012). Cytocompatible antifungal acrylic resin containing silver nanoparticles for dentures. Int. J. Nanomed..

[141] Kamonkhantikul K., Arksornnukit M., Takahashi H. (2017). Antifungal, optical, and mechanical properties of polymethylmethacrylate material incorporated with silanized zinc oxide nanoparticles. Int. J. Nanomed..

[142] Gad M.M., Al-Thobity A.M., Shahin S.Y., Alsaqer B.T., Ali A.A. (2017). Inhibitory effect of zirconium oxide nanoparticles on *Candida albicans* adhesion to repaired polymethyl methacrylate denture bases and interim removable prostheses: A new approach for denture stomatitis prevention. Int. J. Nanomed..

[143] Gowri S., Rajiv Gandhi R., Sundrarajan M. (2014). Structural, Optical, Antibacterial and Antifungal Properties of Zirconia Nanoparticles by Biobased Protocol. J. Mater. Sci. Technol..

[144] Mahmudi A., Varmira K., Jamshidy L. (2020). Determining Efficacy and Minimum Inhibitory Concentrations of a Denture Adhesive Containing Particles and Nanoparticles of Zirconium against *Candida albicans*. J. Evol. Med. Dent. Sci..

[145] Nam K.-Y., Lee C.-H., Lee C.-J. (2012). Antifungal and physical characteristics of modified denture base acrylic incorporated with silver nanoparticles. Gerodontology.

[146] Soliman G.M. (2017). Nanoparticles as safe and effective delivery systems of antifungal agents: Achievements and challenges. Int. J. Pharm..

[147] Renzi D.F., Campos L.D.A., Miranda E.H., Mainardes R.M., Abraham W.-R., Grigoletto D.F., Khalil N.M. (2020). Nanoparticles as a tool for broadening antifungal activities. Curr. Med. Chem..

[148] Al-Maghrabi P.M., Khafagy E.-S., Ghorab M.M., Gad S. (2020). Influence of formulation variables on miconazole nitrate–loaded lipid based nanocarrier for topical delivery. Colloids Surf. B Biointerfaces.

[149] Nagrath M., Sikora A., Graca J., Chinnici J.L., Rahman S.U., Reddy S.G., Ponnusamy S., Maddi A., Arany P.R. (2018). Functionalized prosthetic interfaces using 3D printing: Generating infection-neutralizing prosthesis in dentistry. Mater. Today Commun..

